# Improving large-scale biomass and total alkaloid production of *Dendrobium nobile* Lindl. using a temporary immersion bioreactor system and MeJA elicitation

**DOI:** 10.1186/s13007-022-00843-9

**Published:** 2022-01-22

**Authors:** Benhou Zhang, Zhitao Niu, Chao Li, Zhenyu Hou, Qingyun Xue, Wei Liu, Xiaoyu Ding

**Affiliations:** College of Life Sciences, Nanjing Normal University, Nanjing, 210023 China

**Keywords:** *Dendrobium nobile* Lindl., Temporary immersion bioreactor system, Immersion frequency, MeJA, Bioactive compound

## Abstract

**Background:**

*Dendrobium nobile* Lindl. is an important pharmacopeial plant with medicinal and ornamental value. This study sought to provide a technical means for the large-scale production of total alkaloid in *D. nobile*. Seedlings were cultured in vitro using a temporary immersion bioreactor system (TIBS). The four tested immersion frequencies (min/h; 5/2, 5/4, 5/6, and 5/8) influenced the production of biomass and total alkaloid content. In addition, to compare the effects of different concentrations of the phytohormone methyl jasmonate (MeJA) and treatment time on biomass and total alkaloid accumulation, MeJA was added to the TIBS medium after 50 days. Finally, total alkaloid production in semi-solid system (SSS), TIBS, and TIBS combined with the MeJA system (TIBS-MeJA) were compared.

**Results:**

The best immersion frequency was found to be 5/6 (5 min every 6 h), which ensured appropriate levels of biomass and total alkaloid content in plantlets. The alkaloid content and production level of seedlings were the highest after treatment with 10 μM MeJA separately for 20 and 30 days using TIBS. The maximum content (7.41 mg/g DW) and production level (361.24 mg/L) of total alkaloid on use of TIBS-MeJA were 2.32- and 4.69-fold, respectively, higher in terms of content, and 2.07- and 10.49-fold, respectively, higher in terms of production level than those on using of TIBS (3.20 mg/g DW, 174.34 mg/L) and SSS (1.58 mg/g DW, 34.44 mg/L).

**Conclusions:**

Our results show TIBS-MeJA is suitable for large-scale production of total alkaloid in in vitro seedlings. Therefore, this study provides a technical means for the large-scale production of total alkaloid in *D. nobile.*

## Introduction

*Dendrobium*, which comprises approximately 1200–1500 species, is one of the largest genera in the family Orchidaceae, and there are approximately 80 species within the genus *Dendrobium* in China [1]. *Dendrobium nobile* Lindl is one of the most widespread species within the genus and is an important herb that has many medically important secondary metabolites, including alkaloids, flavonoids, and bioactive polysaccharides [2, 3]. In the ancient Chinese medical book Compendium of Materia Medica, *D. nobile* is described as “the strong body.” Over the past few decades, *D. nobile* has been collected in large quantities because of its high medicinal and ornamental values, resulting in the species becoming increasingly scarce, and active preservation orders have been implemented in several counties. To solve the shortage of *D. nobile*, many researchers are leaning on tissue culture seedlings instead of wild plants [4–6]. However, most of them use a semi-solid system (SSS) with narrow culture space and no effective exchange of gas inside and outside the container.

Alkaloids with complex chemical structures consist of pyrrole, indolizidine, terpenoid alkaloids, amine alkaloids, indole, quinazoline, and others are the most common active compounds in *D. nobile* and are found in all parts of the plant [7]. Modern pharmacology studies have shown that alkaloids can relieve pain, have antipyretic effects, reduce heart rate and blood pressure, slow down respiration, and alleviate barbiturate poisoning [8, 9]. Furthermore, alkaloids have numerous therapeutic activities, including hypoglycemic, anti-cataract, anti-tumor, anti-cell withering, and antioxidant activities; they have also been shown to be effective in treatment of Alzheimer’s disease [10, 11]. However, *D. nobile* alkaloids are mainly obtained from three-year-old plants, which are not only expensive because of their long culture duration but also require a lot of space [12].

Plant tissue culture technology is a rapid and mass propagation method for medicinal plants, and has the potential to increase the yield of secondary metabolites [13]. However, diverse culture systems influenced the propagation of plants and production efficiency of bioactive compounds [14–16]. At present, temporary immersion bioreactor system (TIBS) shows a better performance in plant biomass and bioactive compounds accumulation, especially in officinal plants [17–20]. TIBS is a liquid culture method that allows the explants to contact the medium intermittently, thus renewing the atmosphere and supplying nutrients to meet the growth of plants [21]. This semi-automatic micropropagation system is considered to be an effective method to reduce production costs and labor to a greater degree as compared with those of traditional culture methods, such as semi-solid system (SSS) [22].

Many studies have confirmed that the phytohormone methyl jasmonate (MeJA) can enhance the biosynthesis of secondary metabolites in officinal plants [23–25]. MeJA, as an elicitor, plays an important role in the signal transduction of alkaloids biosynthesis and has been reported to promote the accumulation of metabolites in *Dendrobium* plants [26–29]. In the present study, TIBS and MeJA were used to promote biomass and alkaloid accumulation in *D. nobile* seedlings.

## Results

### Effect of immersion frequency on plantlet biomass and total alkaloid during TIBS culture

**Fig. 1:**
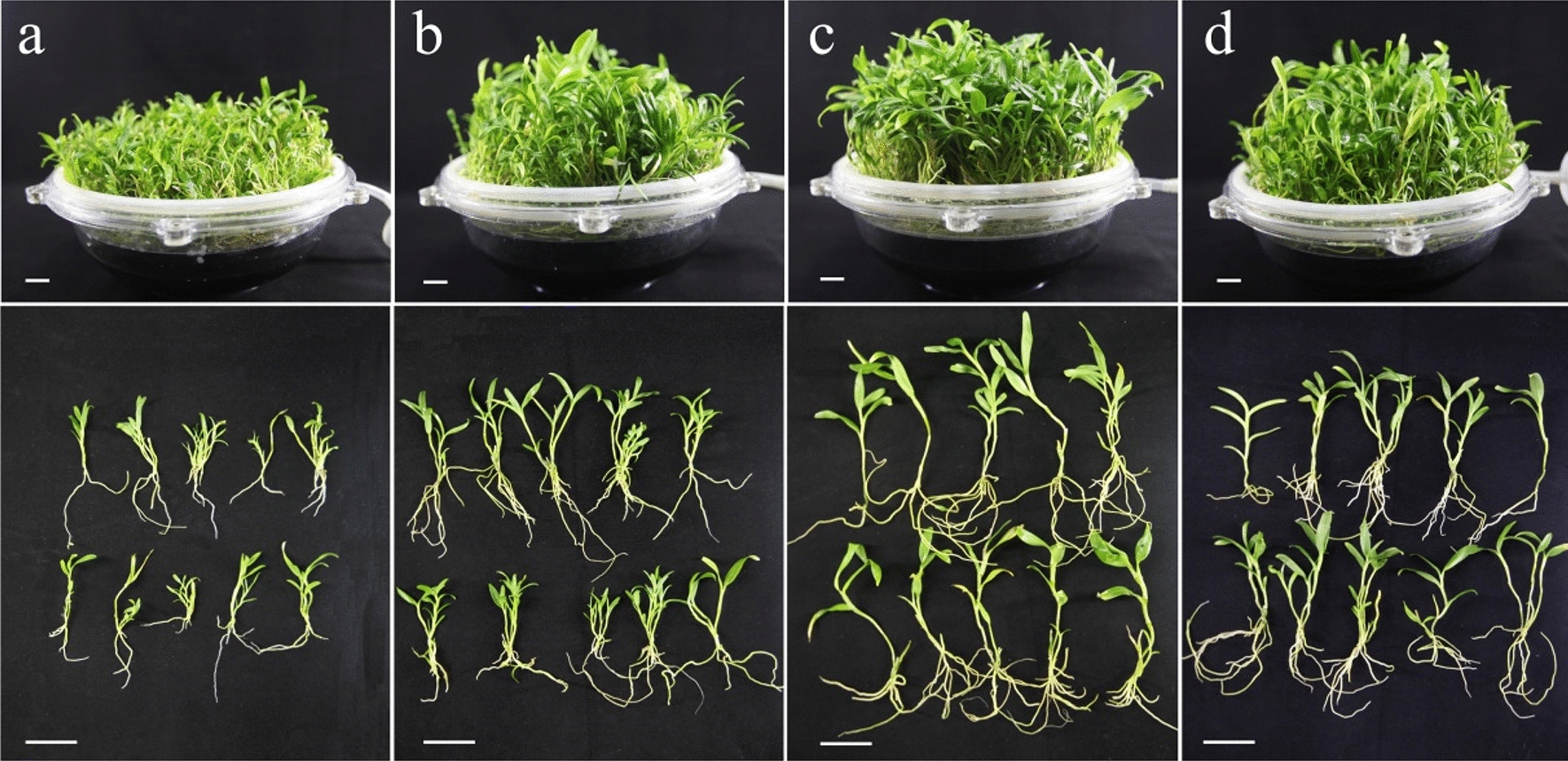
Effect of immersion frequency in TIBS on growth of *D. nobile* for 80 days. **A**–**d** temporary immersion for 5 min every 2 h, every 4 h, every 6 h, and every 8 h respectively. Scale bar = 2 cm

**Fig. 2:**
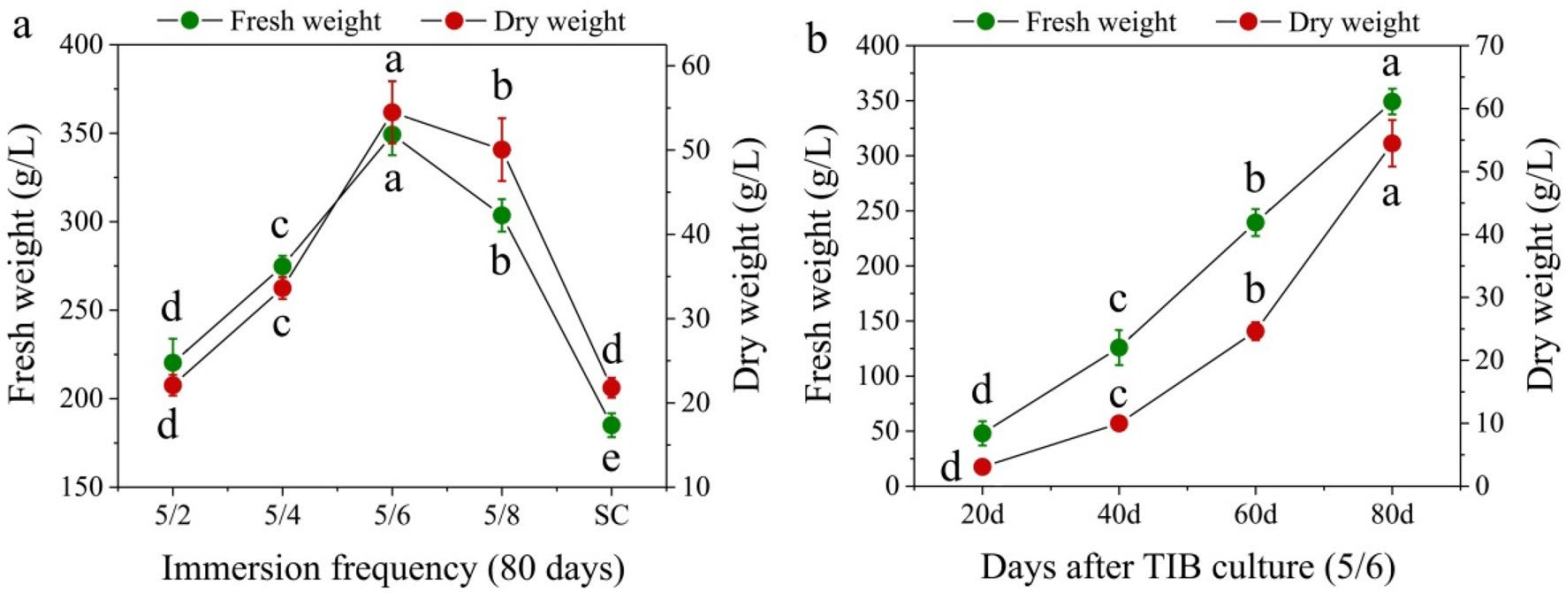
**a** Effect of immersion frequency on biomass accumulation of *D. nobile* after 80 days of TIBS culture. **b** Effect of culture time on biomass accumulation of *D. nobile* at an immersion frequency of 5 min every 6 h of TIBS culture. Data represents the mean ± standard error of three replicates. Bars followed by a different letter denote significant statistical differences (Tukey, P ≤ 0.05)

In this study, four different immersion frequencies (5/2, 5/4, 5/6, and 5/8) were designed using TIBS culture, and a traditional SSS culture was used as a control. After 80 days of growth in the TIBS culture, there were significant differences in the morphology of seedlings under different immersion frequencies, especially under 5/2, and the plantlets were dwarfed and crowded in the tank. The best plantlet morphology was found at an immersion frequency of 5/6, where the plants were taller and the roots showed superior morphology; the second best immersion frequency was 5/8, followed by 5/4, but the two were not obvious (Fig. 1). Figure 2a shows the biomass of seedlings cultured in the reactor for 80 d under different immersion frequencies. The maximum values of fresh weight (349.23 g/L) and dry weight (54.48 g/L) appeared in the immersion frequency of 5/6. For these parameters, the second-best immersion frequency was 5/8, followed by 5/4 and 5/2. When the immersion frequency was fixed at 5/6, the fresh weight and dry weight of the seedlings in the TIBS tank increased with increasing culture time (Fig. 2b).

**Fig. 3:**
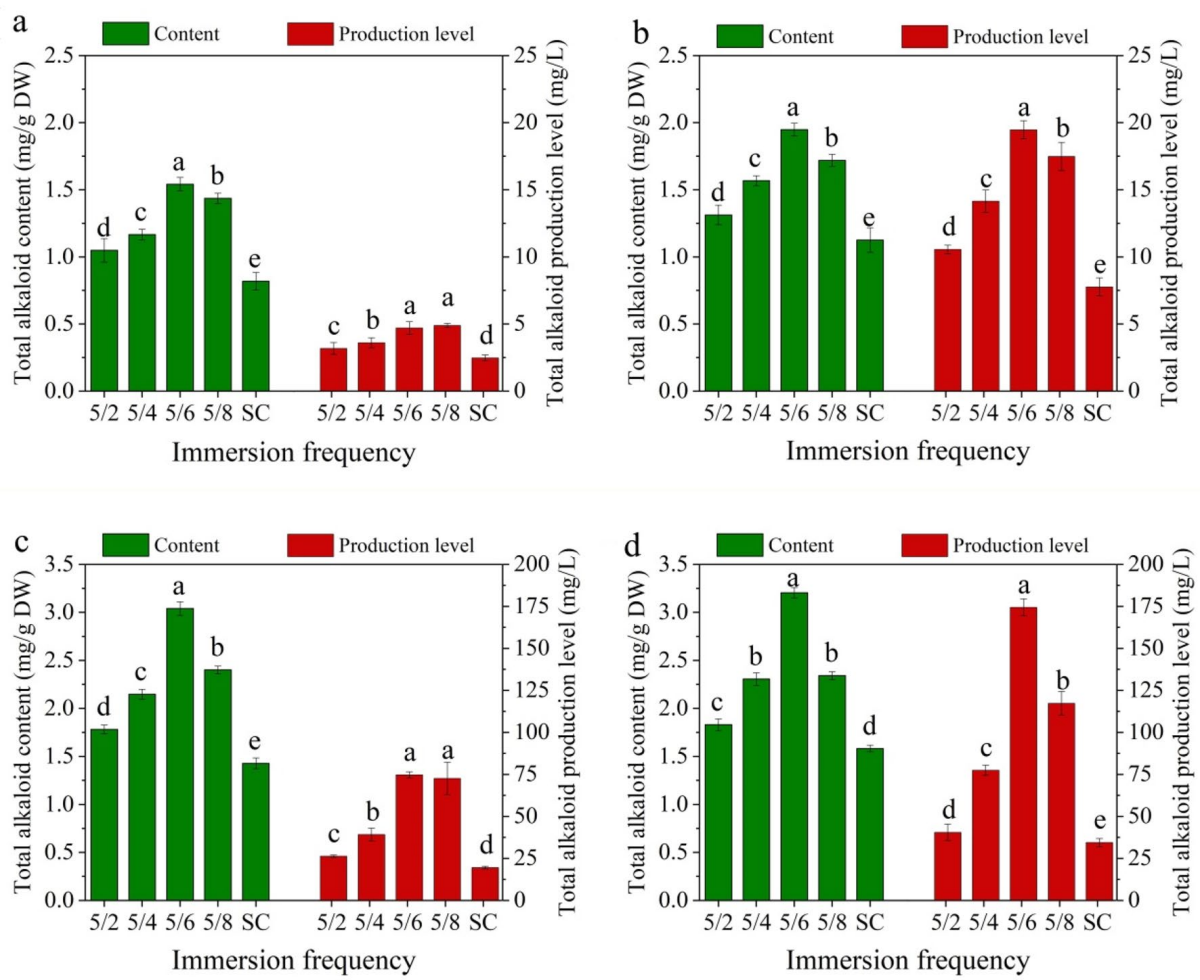
Effect of immersion frequency on total alkaloid accumulation of *D. nobile* after **a** 20 days, **b** 40 days, **c** 60 days and **d** 80 days of culture. SC means semi-solid system culture as a control. Data represents the mean ± standard error of three replicates. Bars followed by a different letter denote significant statistical differences (Tukey, P ≤ 0.05)

Figure 3 shows the effects of immersion frequency and culture duration on the total alkaloid content and production level of plantlets using TIBS culture. The highest total alkaloid content was found at an immersion frequency of 5/6 for all culture durations. The total alkaloid production level of the immersion frequency 5/8 was slightly higher than 5/6 after 20 days of cultivation, but the highest production level was observed at an immersion frequency of 5/6 for other culture durations. Moreover, the total alkaloid content and production level obtained using TIBS culture were significantly higher than those obtained using the traditional SSS culture for all culture durations. As seen in Fig. 3, the highest total alkaloid content (3.20 mg/g DW) and production level (174.44 mg/L) appeared at an immersion frequency of 5/6 after 80 days of cultivation.

### Effect of MeJA concentration during TIBS culture

**Fig. 4:**
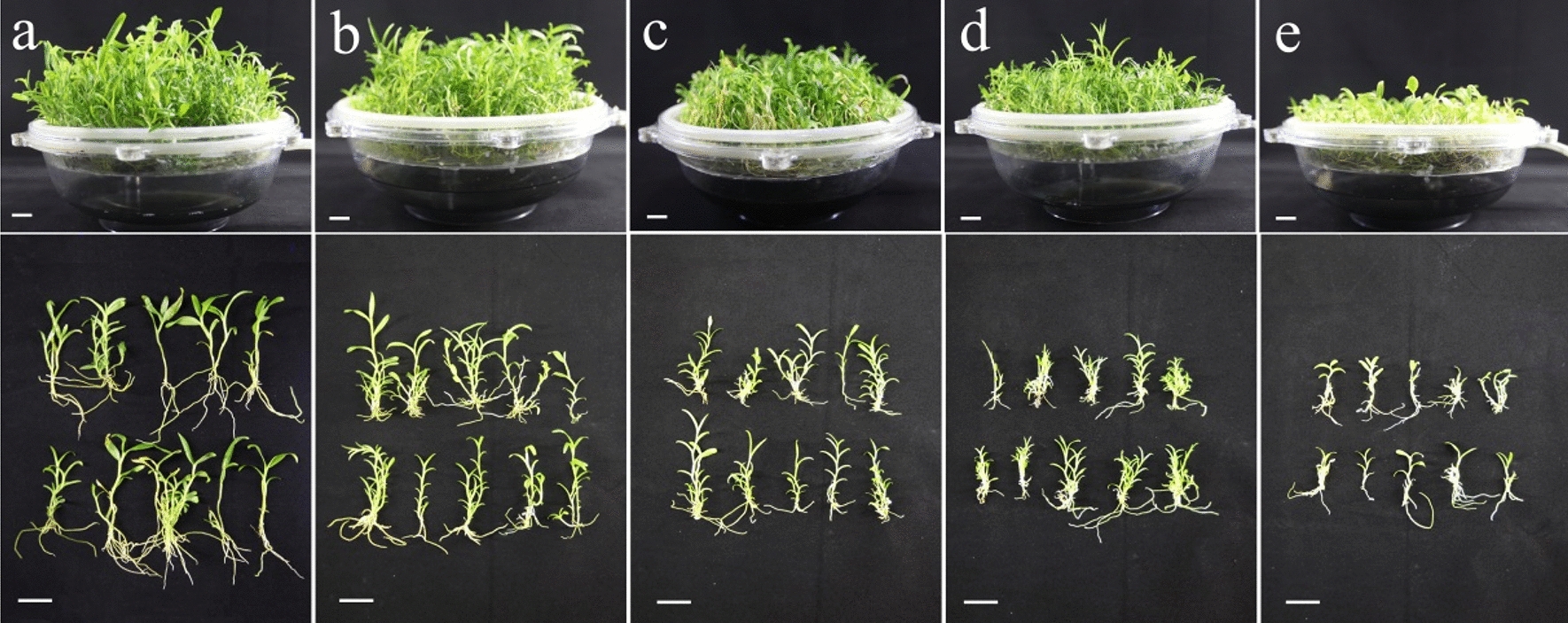
Effect of MeJA concentrations (**a** 0 μM, **b** 5 μM, **c** 10 μM, **d** 15 μM and **e** 20 μM) in TIBS medium on growth of *D. nobile* for 30 days of MeJA treatment. Scale bar = 2 cm

**Fig. 5:**
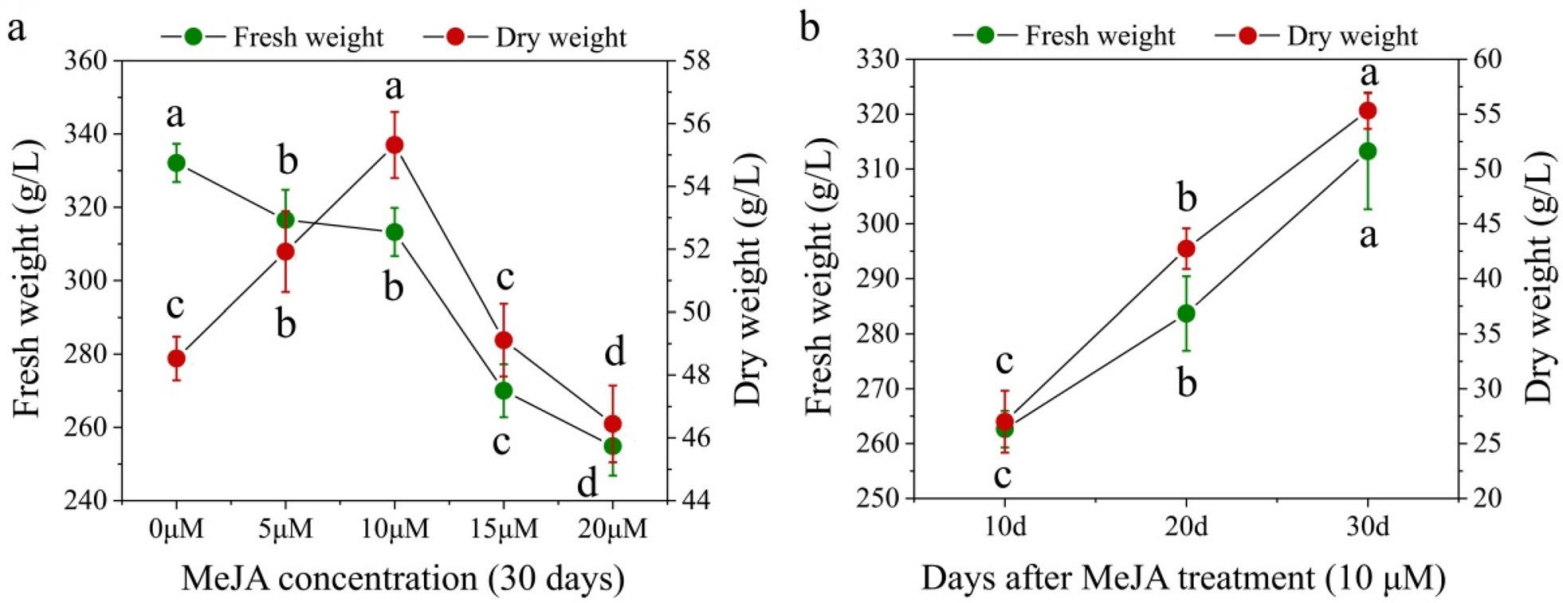
**a** Effect of MeJA concentration on biomass accumulation of *D. nobile* after 30 days of MeJA treatment using TIBS culture. **b** Effect of culture time on biomass accumulation of *D. nobile* at a MeJA concentration of 10 μM using TIBS culture. Data represents the mean ± standard error of three replicates. Bars followed by a different letter denote significant statistical differences (Tukey, P ≤ 0.05)

In this study, different concentrations of MeJA were added to the liquid medium after the plantlets were cultured in TIBS for 50 days. After 30 days of induction culture, different concentrations of MeJA were shown to have significant effects on the growth and proliferation of *D. nobile* plantlets (Fig. 4). Treatment with MeJA resulted in the necrosis of some plantlets, especially at 20 μM MeJA, where almost half of the plantlets were necrotic. Furthermore, from the perspective of individual plants, as the concentration of MeJA increased, plantlets became shorter, with worse root growth, and a small number of plantlets became yellow. The control group without MeJA did not show necrosis and exhibited the best growth, including the roots. Figure 5 shows the fresh and dry weights of seedlings treated with different concentrations of MeJA for 30 days (Fig. 5a) and 10 μM MeJA for different times (Fig. 5b) cultured using TIBS. With MeJA concentrations increasing from 0 to 20 μM (interval of 5 μM), the fresh weight of plantlets decreased gradually. However, the maximum dry weight was obtained at 10 μM MeJA, which was 6.79 g/L higher than the control group (0 μM MeJA) (Fig. 5a).

**Fig. 6:**
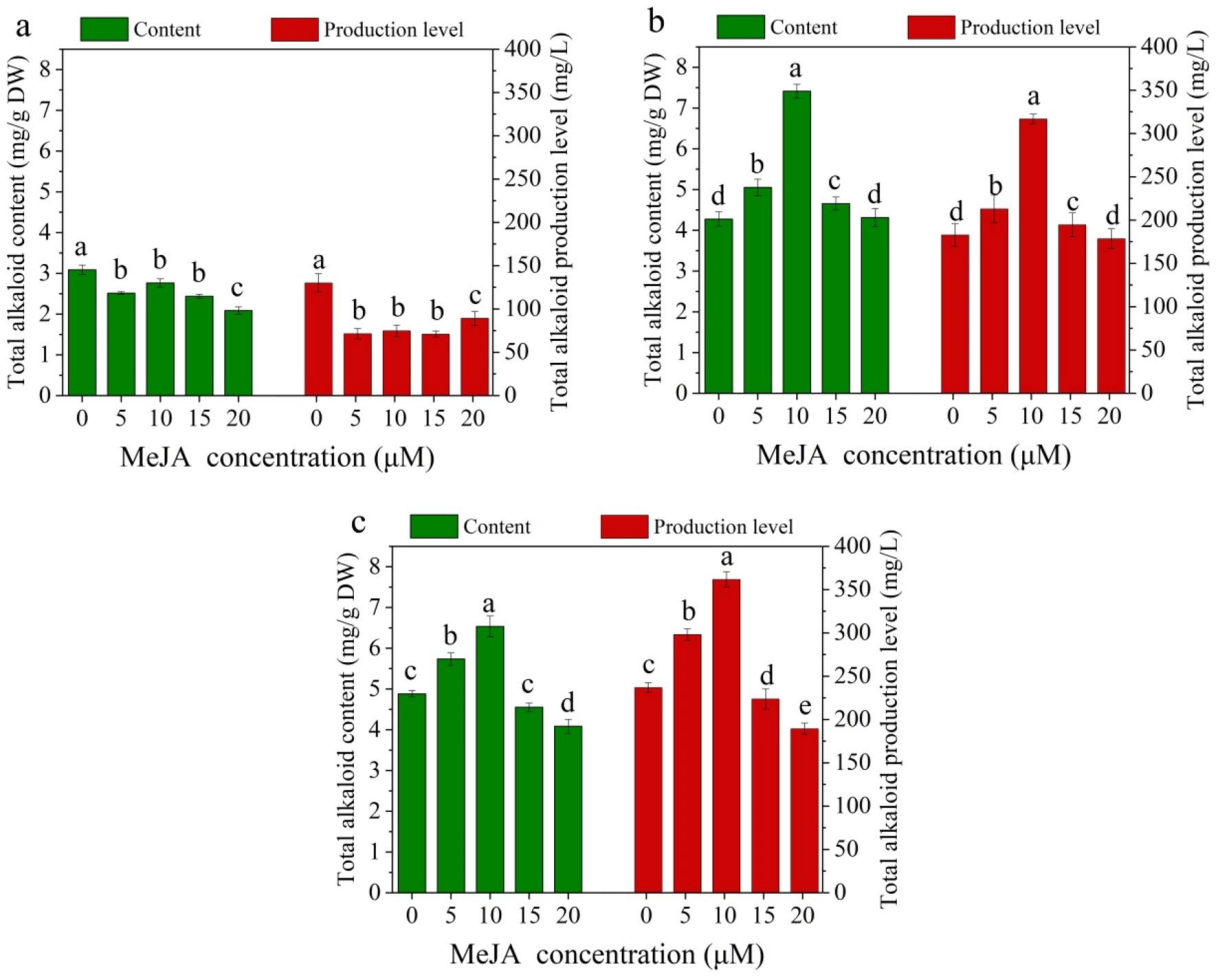
Effect of MeJA concentration on total alkaloid accumulation of *D. nobile* after **a** 10 days, **b** 20 days and **c** 30 days of MeJA treatment. Data represents the mean ± standard error of three replicates. Bars followed by a different letter denote significant statistical differences (Tukey, P ≤ 0.05)

Bioactive compound accumulation in *D. nobile* plantlets cultured using TIBS was significantly affected by MeJA treatment. The total alkaloid content and production level in the MeJA treatment groups were markedly lower than those in the control group after 10 days of MeJA treatment. But, the total alkaloid content and production level were significantly higher than other treatment groups and control group after 20 and 30 days of MeJA treatment in the 10 μM MeJA treatment group, and the maximum content (7.41 mg/g DW) and production level (361.24 mg/L) were observed after 20 and 30 days of MeJA treatment, respectively. However, when the MeJA concentration increased to 20 μM, the total alkaloid content was lower than other treated groups after 10 and 30 days of MeJA treatment, which were 67.62% and 83.65% of the control group, and the production level was lower than other treated groups after 20 and 30 days of MeJA treatment, which were 97.66% and 79.94% of the control group (Fig. 6).

### Comparison of SSS, TIBS, and TIBS-MeJA

The biomass and total alkaloid content of in vitro seedlings grown under the three culture modes were compared to determine the most suitable scheme for the production of alkaloids by *D. nobile*. In all culture modes, the fresh medium was replaced after 50 days of culture, and the MeJA was added to the fresh medium in the type of TIBS combined with the MeJA system (TIBS-MeJA). Table 1 shows that the optimum total alkaloid content, fresh weight, and dry weight of *D. nobile* in different culture systems, and the TIBS cultured seedlings were all significantly higher than those in the traditional SSS culture. Specifically, TIBS-MeJA contained the maximum total alkaloid content (7.41 mg/g DW) after 20 days of MeJA treatment, which was 2.32- and 4.69-fold higher than TIBS and SSS without MeJA after 80 days of culture. It can be calculated from Table 1 that the maximum total alkaloid production level (361.24 mg/L) appeared at TIBS-MeJA after 30 days of MeJA treatment was 2.07- and 10.49-fold higher than TIBS and SSS, respectively. Therefore, we propose that the combination of TIBS and MeJA is an ideal method for the production of alkaloids from *D. nobile* plant tissue culture seedlings.

**Table 1:** Comparison of optimum total alkaloids and biomass production of *D. nobile* in different culture systems

Culture system	Total alkaloid content (mg/g DW)	Fresh weight (g/L)	Dry weight (g/L)	Total culture time (d)
SSS	1.58 ± 0.03d	185.02 ± 6.75d	21.80 ± 0.68c	80
TIBS	3.20 ± 0.05c	349.23 ± 11.63a	54.48 ± 3.69a	80
TIBS-MeJA	7.41 ± 0.17a	283.68 ± 6.76c	42.73 ± 0.85b	70
	6.53 ± 0.26b	313.22 ± 10.57b	55.32 ± 1.65a	80

TIBS-MeJA means seedlings are cultured with MeJA using TIBS

The total culture time was the sum of tissue culture time and MeJA elicitation culture time

Values in each column followed by different letters are significantly different at p ≤ 0.05 as of Post Hoc Multiple Comparisons Test

## Discussion

To mimic natural plant growth conditions, TIBS is designed using a liquid medium to intermittently contact plant tissue to provide nutrition. This system provides an advantageous growth environment for plantlets in liquid culture, including easily absorbed nutrients and effective gas exchange, to ensure the healthy growth of seedlings [30, 31]. In this study, TIBS culture was more conducive to the accumulation of bioactive substances in *D. nobile* plantlets than SSS culture, was consistent with the results of Ashraf et al. [32] and Schumann et al. [33]. TIBS delivers an extremely aerobic environment for plant growth, as it provides forced ventilation through aeration. The immersion frequency is the most significant parameter for system productivity, which not only affects plant growth and micropropagation, but also affects the accumulation of bioactive compounds, especially medicinal plants [34–36]. The results of this study showed that lower immersion frequency (5/6 and 5/8) was beneficial to the accumulation of total alkaloid and biomass in plantlets, whereas higher immersion frequency (5/2 and 5/4) had the opposite effect (Fig. 3). These findings were consistent with those of Ivanov et al. [37] and Malik et al. [38]. The reason for this may be that the higher immersion frequency causes hyperhydricity and malformations of explants to reduce biomass and metabolites, since oxygen concentration in liquid media is often insufficient to meet the respiratory requirements of the submerged tissues [39].

MeJA treatment has significant effects on biomass and bioactive compounds accumulation during plant cell, tissue, and organ cultures [40–44]. Generally, MeJA induces the biosynthesis of alkaloid in plants of the *Dendrobium* genus by up-regulating the expression of alkaloid synthesis related genes [27, 45, 46]. However, the optimal concentration and treatment time of MeJA for maximum yield of active substances varied with the culture systems; for example, 100 μM MeJA showed a maximum level of saponin content in cell suspension culture of *Leucas aspera Spreng* over a period of 18 days [47]. Treatment with 150 μM MeJA for 72 h enhanced camptothecin production in tissue cultures of *Ophiorrhiza mungos* var. *angustifolia* [48], and treatment with 100 μM MeJA for 7 days promoted the production of valerian acid in valerian hairy root cultures of *Valeriana officinalis* [49]. Therefore, it is necessary to screen the MeJA concentration and treatment time to obtain the maximum accumulation of bioactive compounds. In this study, we added different concentrations of MeJA to the TIBS liquid medium that cultured for 50 d. Results showed that the maximum total alkaloid content of plantlets was obtained at 10 μM MeJA after cultured for an additional 20 days. Thus, we can speculate that MeJA enhances the synthesis of alkaloids in in vitro propagated seedlings, as has been shown in previous studies [50, 51]. In this study, we also know that the fresh weight was significantly lower than the control group, even at the lowest MeJA concentration. Plant tissue culture systems have diminished fresh weight after MeJA treatment [52–54], because MeJA can inhibit plant growth by restricting the volume and number of leaf cells and inhibiting mitosis through special patterns. However, the heaviest dry weight appeared at 10 μM MeJA, while the fresh weight was heaviest without MeJA, which may be because MeJA not only promoted the accumulation of the secondary metabolites [55], but also reduced the water content of explants by promoting senescence [56, 57].

*D. nobile* grows for a long time in nature, requiring growth periods of approximately three years; hence, the period of extraction of bioactive compounds from plants is very long [12]. To quickly obtain medicinal plant compounds, plant tissue culture technologies that can effectively shorten the growth cycle have been designed and adopted. For example, plant tissue culture for sustainable valorization of secondary metabolites of *Bryophyllum* sp. [58], and in vitro shoot culture of *Rhododendron fortunei* was used for the commercial production of raw materials for extracting bioactive phytochemicals [59]. Previous studies have reported that the use of bioreactor culture systems and MeJA induction could promote the accumulation of plant bioactive compounds [50, 51, 60, 61]. In the present study, we combined the bioreactor systems with MeJA to efficiently produce alkaloids from *D. nobile* tissue culture seedlings. The results show the TIBS-MeJA was more conducive to the synthesis of alkaloids and could effectively shorten the production cycle, thus reducing production costs. During the culture process of the TIBS, the gas exchange inside and outside of the container could be effectively carried out to ensure the demand for CO_2_ and O_2_ for photosynthesis and respiration [62, 63]. Therefore, tissue culture seedlings grew healthily in TIBS, resulting in more accumulation of alkaloids precursor substances and improvement of the ability of plants to synthesize alkaloids. In addition, the liquid medium used in the TIBS led to the uniform distribution of MeJA as an inducer in the medium [64], which had the ability to continuously promote the synthesis of alkaloids.

## Conclusions

TIBS can be applied to large-scale production of *D. nobile*, and total alkaloid accumulation can be improved by immersion frequency 5/6. Moreover, treatment with MeJA had a high elicitation effect on bioactive compound accumulation in in vitro-cultured *D. nobile* seedlings, and 10 μM MeJA for 20 days of treatment promoted the production of total alkaloid. Thus, we successfully used TIBS-MeJA to obtain the highest total alkaloid content and production level.

## Materials and methods

### Plant materials and preparation

*Dendrobium nobile* seeds were collected from Yunnan Province, China and grown in a greenhouse at Nanjing Normal University. Mature capsules of *D. nobile* obtained through artificial pollination were surface sterilized using 75% alcohol and 10% hydrogen peroxide. Then, the sterile seeds were plated on ½ MS medium (pH 6.0), replenished with 25 g/L sucrose, 80 g/L CW (coconut water), 0.5 mg/L NAA (α-naphthaleneacetic acid), and 7.2 g/L agar. Seeds were cultured in a photoperiod of 10 h light/14 h dark at 25 ± 1 °C for 30 days after 5 days of dark culture, and plantlets (stem length: 2–3 cm) from the seeds were used as plant material for further experiments.

### TIBS and SSS culture of *D. nobile*

**Fig. 7:**
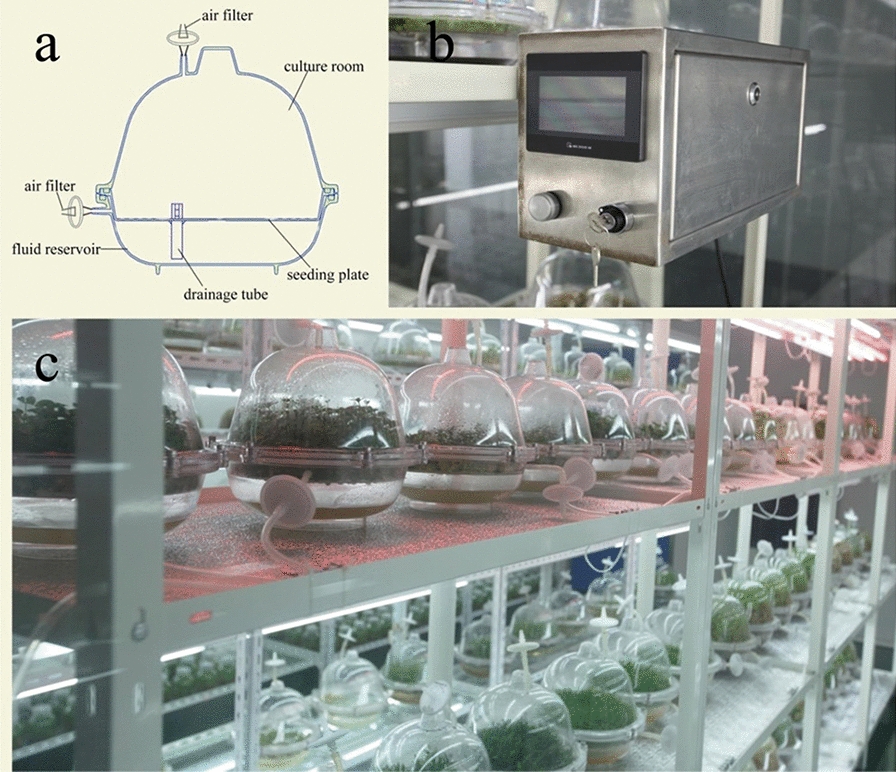
**a** TIBS tank, **b** TIBS controller, **c** a typical culturing array

TIBS was provided by Biofunction Co. Ltd. (Nanjing, China) with a 6.6 L culture tank, which included a controller, culture tank, connecting tube, and air filter (0.22 μm) (Fig. 7). The liquid ½ MS medium (pH 6.0, 1 L) containing 25 g/L sucrose, 0.5 mg/L NAA, and 80 g/L CW, and 300 *D. nobile* plantlets were placed in each container for all treatments. Four immersion frequencies (5/2, 5/4, 5/6, and 5/8) were compared for the biomass and alkaloid content of *D. nobile* seedlings in TIBS. Here, immersion frequencies indicate the time immersed in liquid culture and the interval; “5/2” for example, indicates that the plants were immersed in liquid medium for 5 min every 2 h.

In the SSS experiment, 300 explants were grown in 20 plantlet bottles (each bottle volume 0.5 L) and each bottle contained 40 mL semi-solid medium. SSS medium was added to 7.2 g/L agar as support. All cultures were maintained under a 10 h photoperiod under cool white light (1800 l×) at 25 ± 1 °C for 80 days.

### MeJA elicitation experiments

MeJA (Sigma-Aldrich, 392,707) was dissolved in ethanol to prepare a stock solution and filter-sterilized through a 0.22 μm nylon filter. When the plantlets were cultured in TIBS for 50 days, MeJA was added through aseptic replacement of liquid medium, and the immersion frequency was 5/6. MeJA was used as the elicitor at final concentrations of 5, 10, 15, and 20 μΜ (μmol/L), and liquid medium without MeJA was used as control group. All TIBS cultures were maintained under cool white light (1800 l×) at 25 ± 1 °C for a 10 h photoperiod, and plantlets were harvested after 10, 20, and 30 days of MeJA treatment to determine fresh weight, dry weight, and alkaloid accumulation.

### Determination of biomass and total alkaloid content

The tissue culture seedlings of *D. nobile* were removed from the culture containers and washed with tap water. The fresh weight (FW, g/L), after the water on the plant surface was absorbed with absorbent paper, was obtained using an analytical balance. The dry weight (DW, g/L) was measured after the plant was dried in an oven at 60 °C for 36–48 h to absolute dryness. Dried plant (0.5 g) was ground into powder using a mortar, ammonia solution was added, and the mixture was allowed to stand for 0.5 h. Following this, the mixed liquids were poured into a 50 mL flask, and 25 mL of chloroform was added for extraction. The chloroform in the flask was dried with a rotary evaporator after being maintained in a water bath at 70 °C for 2.5 h, and then 5 mL of chloroform was poured into the flask to dissolve the dry residue, and then 2 mL of chloroform extract was aspirated and chloroform was added to the chloroform extract to 10 mL. Potassium hydrogen phthalate buffer (5 mL, pH 4.5) and 2 mL of 0.04% (w/v) bromocresol green solution were mixed and poured into the chloroform extract. The mixture was shaken vigorously for 3 min and allowed to stand for 0.5 h, and then 1 mL alkaline alcohol (0.01 mol/L NaOH) was added to 5 mL lower fractions for analysis. The absorbance value was determined using a spectrophotometer at 620 nm, and then the total alkaloid content was calculated using a standard curve equation: y = 0.063x + 0.027, (R^2^ = 0.996) (y and x are the absorbance and content of dendrobine, respectively), which was obtained with dendrobine as the reference standard. The total alkaloid content and production level were calculated using the following formula: content (mg/g DW) = dendrobine (mg) × 5/0.5 g and production level (mg/L) = DW (g/L) × content (mg/g DW) [27, 52].

## Data Availability

The datasets used and/or analyzed during the current study are available from the corresponding authors upon reasonable request.
